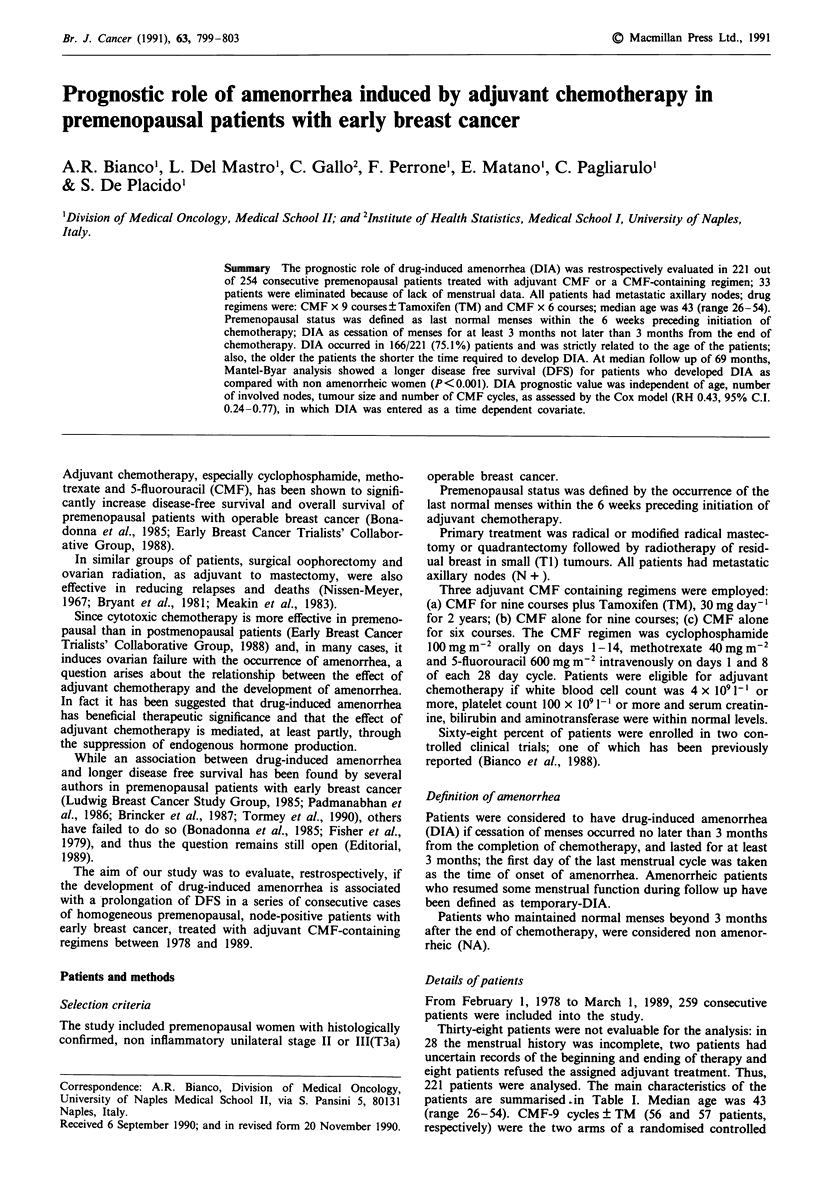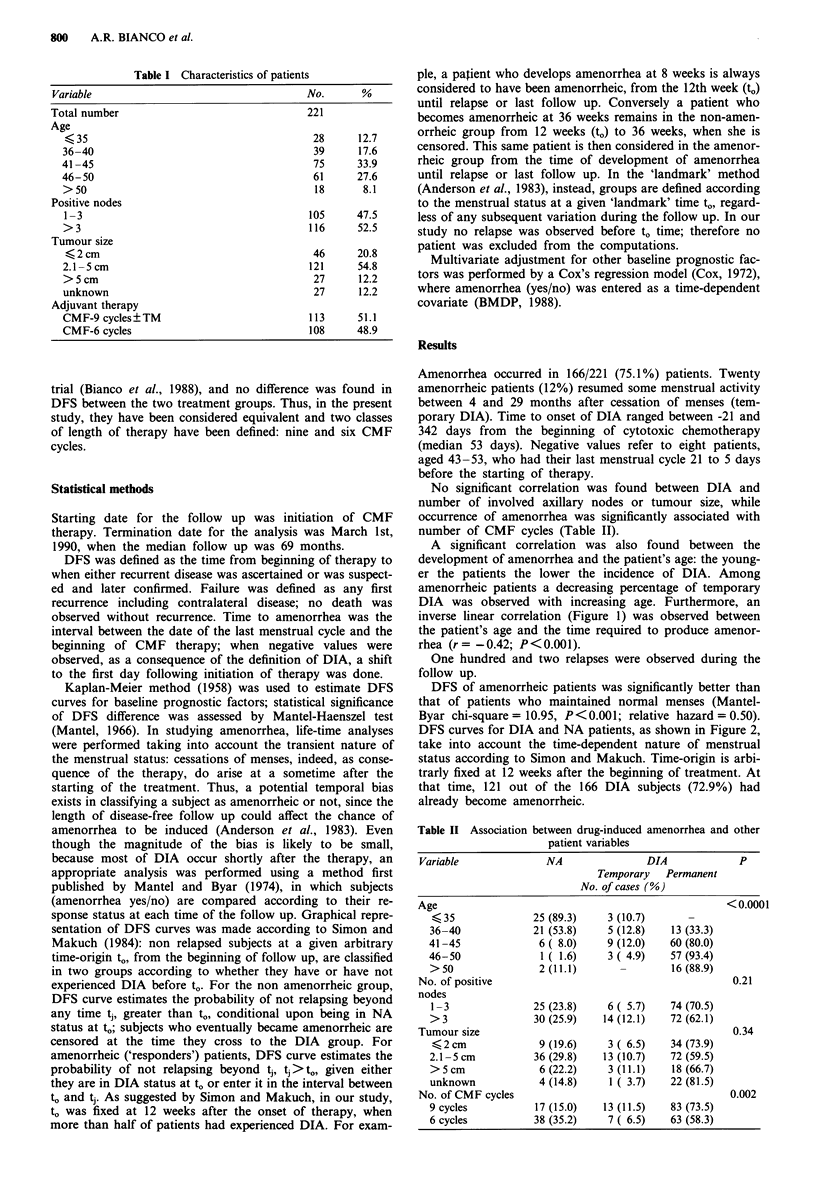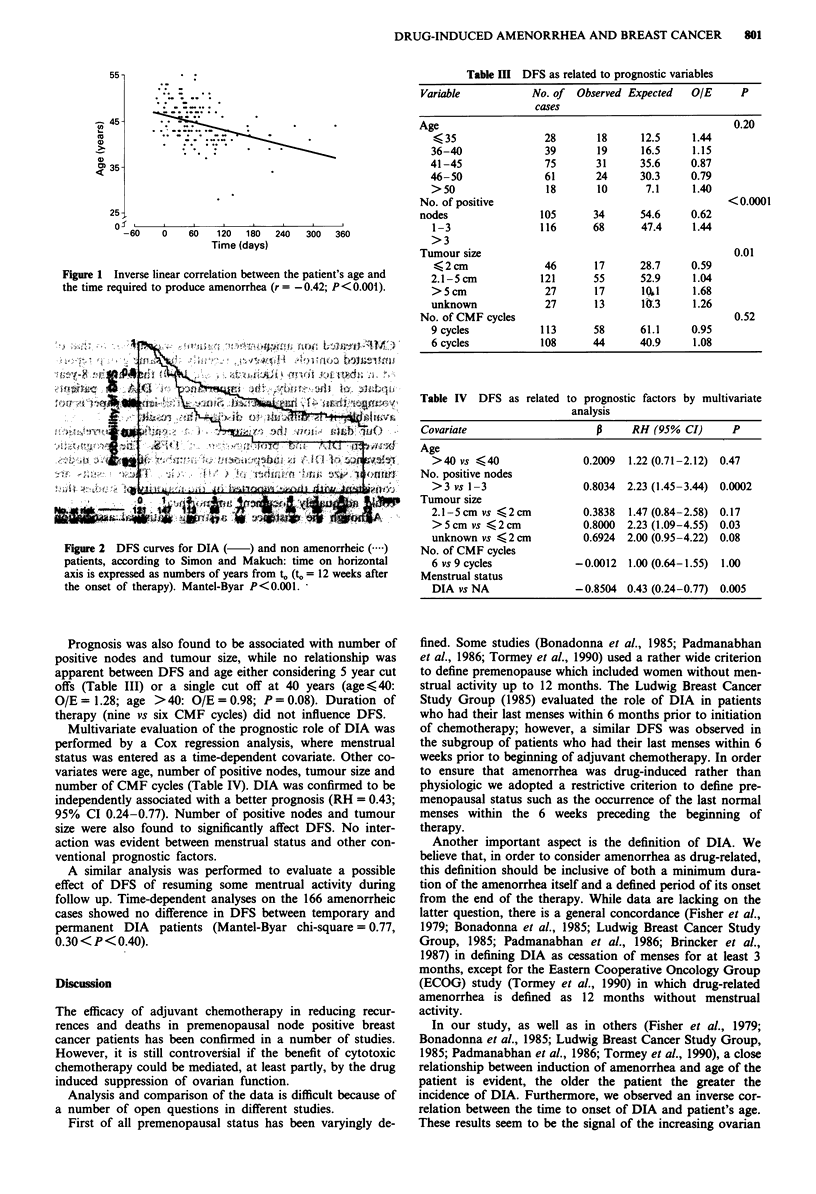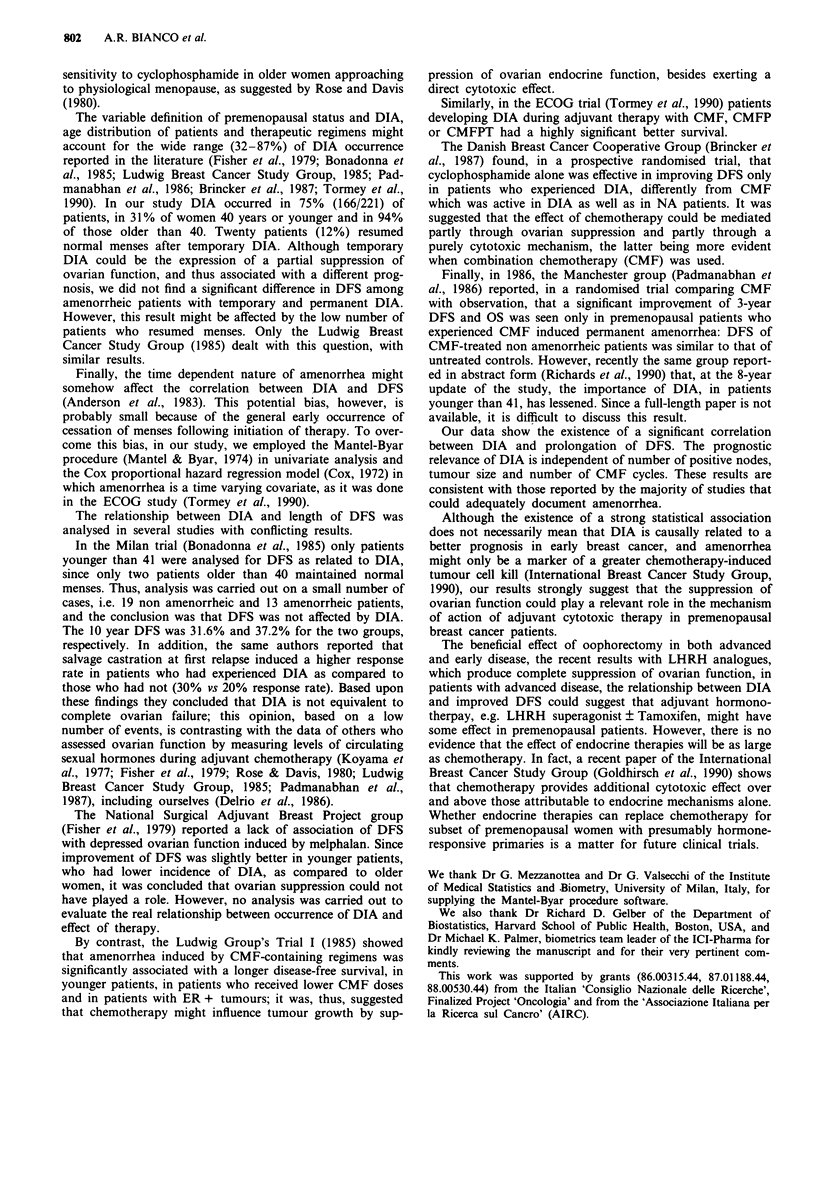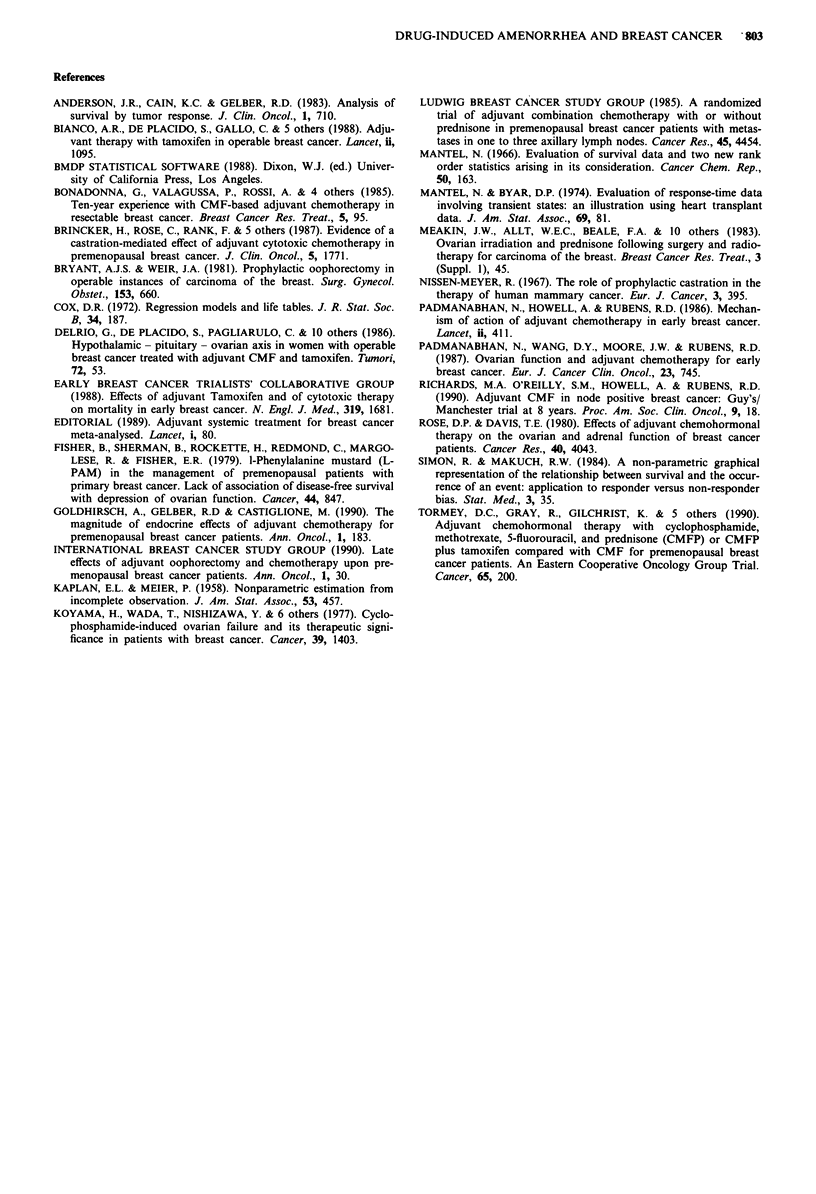# Prognostic role of amenorrhea induced by adjuvant chemotherapy in premenopausal patients with early breast cancer.

**DOI:** 10.1038/bjc.1991.177

**Published:** 1991-05

**Authors:** A. R. Bianco, L. Del Mastro, C. Gallo, F. Perrone, E. Matano, C. Pagliarulo, S. De Placido

**Affiliations:** Division of Medical Oncology, Medical School II, University of Naples, Italy.

## Abstract

The prognostic role of drug-induced amenorrhea (DIA) was restrospectively evaluated in 221 out of 254 consecutive premenopausal patients treated with adjuvant CMF or a CMF-containing regimen; 33 patients were eliminated because of lack of menstrual data. All patients had metastatic axillary nodes; drug regimens were: CMF x 9 courses +/- Tamoxifen (TM) and CMF x 6 courses; median age was 43 (range 26-54). Premenopausal status was defined as last normal menses within the 6 weeks preceding initiation of chemotherapy: DIA as cessation of menses for at least 3 months not later than 3 months from the end of chemotherapy. DIA occurred in 166,221 (75.1%) patients and was strictly related to the age of the patients; also, the older the patients the shorter the time required to develop DIA. At median follow up of 69 months, Mantel-Byar analysis showed a longer disease free survival (DFS) for patients who developed DIA as compared with non amenorrheic women (P less than 0.001). DIA prognostic value was independent of age, number of involved nodes, tumour size and number of CMF cycles, as assessed by the Cox model (RH 0.43, 95% C.I. 0.24-0.77), in which DIA was entered as a time dependent covariate.


Br. J. Cancer (1991), 63, 799-803 © Macmillan Press Ltd., 1991

### Prognostic role of amenorrhea induced by adjuvant chemotherapy in premenopausal patients with early breast cancer

A.R. Bianco[1], L. Del Mastro[1], C. Gallo[2], F. Perrone[1], E. Matano[1], C. Pagliarulo[1] & S. De Placido[1]

[1]*Division of Medical Oncology, Medical School II; and* [2]*Institute of Health Statistics, Medical School I, University of Naples, Italy.*

**Summary** The prognostic role of drug-induced amenorrhea (DIA) was restrospectively evaluated in 221 out of 254 consecutive premenopausal patients treated with adjuvant CMF or a CMF-containing regimen; 33 patients were eliminated because of lack of menstrual data. All patients had metastatic axillary nodes; drug regimens were: CMF × 9 courses ± Tamoxifen (TM) and CMF × 6 courses; median age was 43 (range 26-54). Premenopausal status was defined as last normal menses within the 6 weeks preceding initiation of chemotherapy; DIA as cessation of menses for at least 3 months not later than 3 months from the end of chemotherapy. DIA occurred in 166/221 (75.1%) patients and was strictly related to the age of the patients; also, the older the patients the shorter the time required to develop DIA. At median follow up of 69 months, Mantel-Byar analysis showed a longer disease free survival (DFS) for patients who developed DIA as compared with non amenorrheic women ($P < 0.001$). DIA prognostic value was independent of age, number of involved nodes, tumour size and number of CMF cycles, as assessed by the Cox model (RH 0.43, 95% C.I. 0.24-0.77), in which DIA was entered as a time dependent covariate.

Adjuvant chemotherapy, especially cyclophosphamide, methotrexate and 5-fluorouracil (CMF), has been shown to significantly increase disease-free survival and overall survival of premenopausal patients with operable breast cancer (Bonadonna *et al.*, 1985; Early Breast Cancer Trialists' Collaborative Group, 1988).

In similar groups of patients, surgical oophorectomy and ovarian radiation, as adjuvant to mastectomy, were also effective in reducing relapses and deaths (Nissen-Meyer, 1967; Bryant *et al.*, 1981; Meakin *et al.*, 1983).

Since cytotoxic chemotherapy is more effective in premenopausal than in postmenopausal patients (Early Breast Cancer Trialists' Collaborative Group, 1988) and, in many cases, it induces ovarian failure with the occurrence of amenorrhea, a question arises about the relationship between the effect of adjuvant chemotherapy and the development of amenorrhea. In fact it has been suggested that drug-induced amenorrhea has beneficial therapeutic significance and that the effect of adjuvant chemotherapy is mediated, at least partly, through the suppression of endogenous hormone production.

While an association between drug-induced amenorrhea and longer disease free survival has been found by several authors in premenopausal patients with early breast cancer (Ludwig Breast Cancer Study Group, 1985; Padmanabhan *et al.*, 1986; Brincker *et al.*, 1987; Tormey *et al.*, 1990), others have failed to do so (Bonadonna *et al.*, 1985; Fisher *et al.*, 1979), and thus the question remains still open (Editorial, 1989).

The aim of our study was to evaluate, restrospectively, if the development of drug-induced amenorrhea is associated with a prolongation of DFS in a series of consecutive cases of homogeneous premenopausal, node-positive patients with early breast cancer, treated with adjuvant CMF-containing regimens between 1978 and 1989.

### Patients and methods

### *Selection criteria*

The study included premenopausal women with histologically confirmed, non inflammatory unilateral stage II or III(T3a) operable breast cancer.

Premenopausal status was defined by the occurrence of the last normal menses within the 6 weeks preceding initiation of adjuvant chemotherapy.

Primary treatment was radical or modified radical mastectomy or quadrantectomy followed by radiotherapy of residual breast in small (T1) tumours. All patients had metastatic axillary nodes (N + ).

Three adjuvant CMF containing regimens were employed: (a) CMF for nine courses plus Tamoxifen (TM), 30 mg day$^{-1}$ for 2 years; (b) CMF alone for nine courses; (c) CMF alone for six courses. The CMF regimen was cyclophosphamide 100 mg m$^{-2}$ orally on days 1-14, methotrexate 40 mg m$^{-2}$ and 5-fluorouracil 600 mg m$^{-2}$ intravenously on days 1 and 8 of each 28 day cycle. Patients were eligible for adjuvant chemotherapy if white blood cell count was $4 \times 10^9 l^{-1}$ or more, platelet count $100 \times 10^9 l^{-1}$ or more and serum creatinine, bilirubin and aminotransferase were within normal levels.

Sixty-eight percent of patients were enrolled in two controlled clinical trials; one of which has been previously reported (Bianco *et al.*, 1988).

### *Definition of amenorrhea*

Patients were considered to have drug-induced amenorrhea (DIA) if cessation of menses occurred no later than 3 months from the completion of chemotherapy, and lasted for at least 3 months; the first day of the last menstrual cycle was taken as the time of onset of amenorrhea. Amenorrheic patients who resumed some menstrual function during follow up have been defined as temporary-DIA.

Patients who maintained normal menses beyond 3 months after the end of chemotherapy, were considered non amenorrheic (NA).

### *Details of patients*

From February 1, 1978 to March 1, 1989, 259 consecutive patients were included into the study.

Thirty-eight patients were not evaluable for the analysis: in 28 the menstrual history was incomplete, two patients had uncertain records of the beginning and ending of therapy and eight patients refused the assigned adjuvant treatment. Thus, 221 patients were analysed. The main characteristics of the patients are summarised in Table I. Median age was 43 (range 26-54). CMF-9 cycles ± TM (56 and 57 patients, respectively) were the two arms of a randomised controlled

Correspondence: A.R. Bianco, Division of Medical Oncology, University of Naples Medical School II, via S. Pansini 5, 80131 Naples, Italy.
Received 6 September 1990; and in revised form 20 November 1990.

Table I Characteristics of patients

| Variable | No. | % |
|---|---|---|
| Total number | 221 | |
| Age | | |
| ⩽35 | 28 | 12.7 |
| 36–40 | 39 | 17.6 |
| 41–45 | 75 | 33.9 |
| 46–50 | 61 | 27.6 |
| >50 | 18 | 8.1 |
| Positive nodes | | |
| 1–3 | 105 | 47.5 |
| >3 | 116 | 52.5 |
| Tumour size | | |
| ⩽2 cm | 46 | 20.8 |
| 2.1–5 cm | 121 | 54.8 |
| >5 cm | 27 | 12.2 |
| unknown | 27 | 12.2 |
| Adjuvant therapy | | |
| CMF-9 cycles±TM | 113 | 51.1 |
| CMF-6 cycles | 108 | 48.9 |

trial (Bianco *et al.*, 1988), and no difference was found in DFS between the two treatment groups. Thus, in the present study, they have been considered equivalent and two classes of length of therapy have been defined: nine and six CMF cycles.

### Statistical methods

Starting date for the follow up was initiation of CMF therapy. Termination date for the analysis was March 1st, 1990, when the median follow up was 69 months.

DFS was defined as the time from beginning of therapy to when either recurrent disease was ascertained or was suspected and later confirmed. Failure was defined as any first recurrence including contralateral disease; no death was observed without recurrence. Time to amenorrhea was the interval between the date of the last menstrual cycle and the beginning of CMF therapy; when negative values were observed, as a consequence of the definition of DIA, a shift to the first day following initiation of therapy was done.

Kaplan-Meier method (1958) was used to estimate DFS curves for baseline prognostic factors; statistical significance of DFS difference was assessed by Mantel-Haenszel test (Mantel, 1966). In studying amenorrhea, life-time analyses were performed taking into account the transient nature of the menstrual status: cessations of menses, indeed, as consequence of the therapy, do arise at a sometime after the starting of the treatment. Thus, a potential temporal bias exists in classifying a subject as amenorrheic or not, since the length of disease-free follow up could affect the chance of amenorrhea to be induced (Anderson *et al.*, 1983). Even though the magnitude of the bias is likely to be small, because most of DIA occur shortly after the therapy, an appropriate analysis was performed using a method first published by Mantel and Byar (1974), in which subjects (amenorrhea yes/no) are compared according to their response status at each time of the follow up. Graphical representation of DFS curves was made according to Simon and Makuch (1984): non relapsed subjects at a given arbitrary time-origin $t_o$, from the beginning of follow up, are classified in two groups according to whether they have or have not experienced DIA before $t_o$. For the non amenorrheic group, DFS curve estimates the probability of not relapsing beyond any time $t_j$, greater than $t_o$, conditional upon being in NA status at $t_o$; subjects who eventually became amenorrheic are censored at the time they cross to the DIA group. For amenorrheic ('responders') patients, DFS curve estimates the probability of not relapsing beyond $t_j$, $t_j > t_o$, given either they are in DIA status at $t_o$ or enter it in the interval between $t_o$ and $t_j$. As suggested by Simon and Makuch, in our study, $t_o$ was fixed at 12 weeks after the onset of therapy, when more than half of patients had experienced DIA. For example, a patient who develops amenorrhea at 8 weeks is always considered to have been amenorrheic, from the 12th week ($t_o$) until relapse or last follow up. Conversely a patient who becomes amenorrheic at 36 weeks remains in the non-amenorrheic group from 12 weeks ($t_o$) to 36 weeks, when she is censored. This same patient is then considered in the amenorrheic group from the time of development of amenorrhea until relapse or last follow up. In the 'landmark' method (Anderson *et al.*, 1983), instead, groups are defined according to the menstrual status at a given 'landmark' time $t_o$, regardless of any subsequent variation during the follow up. In our study no relapse was observed before $t_o$ time; therefore no patient was excluded from the computations.

Multivariate adjustment for other baseline prognostic factors was performed by a Cox's regression model (Cox, 1972), where amenorrhea (yes/no) was entered as a time-dependent covariate (BMDP, 1988).

### Results

Amenorrhea occurred in 166/221 (75.1%) patients. Twenty amenorrheic patients (12%) resumed some menstrual activity between 4 and 29 months after cessation of menses (temporary DIA). Time to onset of DIA ranged between -21 and 342 days from the beginning of cytotoxic chemotherapy (median 53 days). Negative values refer to eight patients, aged 43–53, who had their last menstrual cycle 21 to 5 days before the starting of therapy.

No significant correlation was found between DIA and number of involved axillary nodes or tumour size, while occurrence of amenorrhea was significantly associated with number of CMF cycles (Table II).

A significant correlation was also found between the development of amenorrhea and the patient's age: the younger the patients the lower the incidence of DIA. Among amenorrheic patients a decreasing percentage of temporary DIA was observed with increasing age. Furthermore, an inverse linear correlation (Figure 1) was observed between the patient's age and the time required to produce amenorrhea ($r = -0.42$; $P < 0.001$).

One hundred and two relapses were observed during the follow up.

DFS of amenorrheic patients was significantly better than that of patients who maintained normal menses (Mantel-Byar chi-square = 10.95, $P < 0.001$; relative hazard = 0.50). DFS curves for DIA and NA patients, as shown in Figure 2, take into account the time-dependent nature of menstrual status according to Simon and Makuch. Time-origin is arbitrarly fixed at 12 weeks after the beginning of treatment. At that time, 121 out of the 166 DIA subjects (72.9%) had already become amenorrheic.

Table II Association between drug-induced amenorrhea and other patient variables

| Variable | NA | DIA Temporary | DIA Permanent | P |
|---|---|---|---|---|
| | No. of cases (%) | | | |
| Age | | | | <0.0001 |
| ⩽35 | 25 (89.3) | 3 (10.7) | – | |
| 36–40 | 21 (53.8) | 5 (12.8) | 13 (33.3) | |
| 41–45 | 6 ( 8.0) | 9 (12.0) | 60 (80.0) | |
| 46–50 | 1 ( 1.6) | 3 ( 4.9) | 57 (93.4) | |
| >50 | 2 (11.1) | – | 16 (88.9) | |
| No. of positive nodes | | | | 0.21 |
| 1–3 | 25 (23.8) | 6 ( 5.7) | 74 (70.5) | |
| >3 | 30 (25.9) | 14 (12.1) | 72 (62.1) | |
| Tumour size | | | | 0.34 |
| ⩽2 cm | 9 (19.6) | 3 ( 6.5) | 34 (73.9) | |
| 2.1–5 cm | 36 (29.8) | 13 (10.7) | 72 (59.5) | |
| >5 cm | 6 (22.2) | 3 (11.1) | 18 (66.7) | |
| unknown | 4 (14.8) | 1 ( 3.7) | 22 (81.5) | |
| No. of CMF cycles | | | | 0.002 |
| 9 cycles | 17 (15.0) | 13 (11.5) | 83 (73.5) | |
| 6 cycles | 38 (35.2) | 7 ( 6.5) | 63 (58.3) | |

**Figure 1** Inverse linear correlation between the patient's age and the time required to produce amenorrhea ($r = -0.42$; $P<0.001$).

**Figure 2** DFS curves for DIA (——) and non amenorrheic (····) patients, according to Simon and Makuch: time on horizontal axis is expressed as numbers of years from $t_0$ ($t_0$ = 12 weeks after the onset of therapy). Mantel-Byar $P<0.001$.

**Table III** DFS as related to prognostic variables

| *Variable* | *No. of cases* | *Observed* | *Expected* | *O/E* | *P* |
|---|---|---|---|---|---|
| Age | | | | | 0.20 |
| ⩽35 | 28 | 18 | 12.5 | 1.44 | |
| 36–40 | 39 | 19 | 16.5 | 1.15 | |
| 41–45 | 75 | 31 | 35.6 | 0.87 | |
| 46–50 | 61 | 24 | 30.3 | 0.79 | |
| >50 | 18 | 10 | 7.1 | 1.40 | |
| No. of positive | | | | | <0.0001 |
| nodes | 105 | 34 | 54.6 | 0.62 | |
| 1–3 | 116 | 68 | 47.4 | 1.44 | |
| >3 | | | | | |
| Tumour size | | | | | 0.01 |
| ⩽2 cm | 46 | 17 | 28.7 | 0.59 | |
| 2.1–5 cm | 121 | 55 | 52.9 | 1.04 | |
| >5 cm | 27 | 17 | 10.1 | 1.68 | |
| unknown | 27 | 13 | 10.3 | 1.26 | |
| No. of CMF cycles | | | | | 0.52 |
| 9 cycles | 113 | 58 | 61.1 | 0.95 | |
| 6 cycles | 108 | 44 | 40.9 | 1.08 | |

**Table IV** DFS as related to prognostic factors by multivariate analysis

| *Covariate* | *β* | *RH (95% CI)* | *P* |
|---|---|---|---|
| Age | | | |
| >40 *vs* ⩽40 | 0.2009 | 1.22 (0.71–2.12) | 0.47 |
| No. positive nodes | | | |
| >3 *vs* 1–3 | 0.8034 | 2.23 (1.45–3.44) | 0.0002 |
| Tumour size | | | |
| 2.1–5 cm *vs* ⩽2 cm | 0.3838 | 1.47 (0.84–2.58) | 0.17 |
| >5 cm *vs* ⩽2 cm | 0.8000 | 2.23 (1.09–4.55) | 0.03 |
| unknown *vs* ⩽2 cm | 0.6924 | 2.00 (0.95–4.22) | 0.08 |
| No. of CMF cycles | | | |
| 6 *vs* 9 cycles | −0.0012 | 1.00 (0.64–1.55) | 1.00 |
| Menstrual status | | | |
| DIA *vs* NA | −0.8504 | 0.43 (0.24–0.77) | 0.005 |

Prognosis was also found to be associated with number of positive nodes and tumour size, while no relationship was apparent between DFS and age either considering 5 year cut offs (Table III) or a single cut off at 40 years (age⩽40: O/E = 1.28; age >40: O/E = 0.98; $P = 0.08$). Duration of therapy (nine *vs* six CMF cycles) did not influence DFS.

Multivariate evaluation of the prognostic role of DIA was performed by a Cox regression analysis, where menstrual status was entered as a time-dependent covariate. Other covariates were age, number of positive nodes, tumour size and number of CMF cycles (Table IV). DIA was confirmed to be independently associated with a better prognosis (RH = 0.43; 95% CI 0.24–0.77). Number of positive nodes and tumour size were also found to significantly affect DFS. No interaction was evident between menstrual status and other conventional prognostic factors.

A similar analysis was performed to evaluate a possible effect of DFS of resuming some mentrual activity during follow up. Time-dependent analyses on the 166 amenorrheic cases showed no difference in DFS between temporary and permanent DIA patients (Mantel-Byar chi-square = 0.77, $0.30<P<0.40$).

### Discussion

The efficacy of adjuvant chemotherapy in reducing recurrences and deaths in premenopausal node positive breast cancer patients has been confirmed in a number of studies. However, it is still controversial if the benefit of cytotoxic chemotherapy could be mediated, at least partly, by the drug induced suppression of ovarian function.

Analysis and comparison of the data is difficult because of a number of open questions in different studies.

First of all premenopausal status has been varyingly defined. Some studies (Bonadonna *et al.*, 1985; Padmanabhan *et al.*, 1986; Tormey *et al.*, 1990) used a rather wide criterion to define premenopause which included women without menstrual activity up to 12 months. The Ludwig Breast Cancer Study Group (1985) evaluated the role of DIA in patients who had their last menses within 6 months prior to initiation of chemotherapy; however, a similar DFS was observed in the subgroup of patients who had their last menses within 6 weeks prior to beginning of adjuvant chemotherapy. In order to ensure that amenorrhea was drug-induced rather than physiologic we adopted a restrictive criterion to define premenopausal status such as the occurrence of the last normal menses within the 6 weeks preceding the beginning of therapy.

Another important aspect is the definition of DIA. We believe that, in order to consider amenorrhea as drug-related, this definition should be inclusive of both a minimum duration of the amenorrhea itself and a defined period of its onset from the end of the therapy. While data are lacking on the latter question, there is a general concordance (Fisher *et al.*, 1979; Bonadonna *et al.*, 1985; Ludwig Breast Cancer Study Group, 1985; Padmanabhan *et al.*, 1986; Brincker *et al.*, 1987) in defining DIA as cessation of menses for at least 3 months, except for the Eastern Cooperative Oncology Group (ECOG) study (Tormey *et al.*, 1990) in which drug-related amenorrhea is defined as 12 months without menstrual activity.

In our study, as well as in others (Fisher *et al.*, 1979; Bonadonna *et al.*, 1985; Ludwig Breast Cancer Study Group, 1985; Padmanabhan *et al.*, 1986; Tormey *et al.*, 1990), a close relationship between induction of amenorrhea and age of the patient is evident, the older the patient the greater the incidence of DIA. Furthermore, we observed an inverse correlation between the time to onset of DIA and patient's age. These results seem to be the signal of the increasing ovarian sensitivity to cyclophosphamide in older women approaching to physiological menopause, as suggested by Rose and Davis (1980).

The variable definition of premenopausal status and DIA, age distribution of patients and therapeutic regimens might account for the wide range (32–87%) of DIA occurrence reported in the literature (Fisher *et al.*, 1979; Bonadonna *et al.*, 1985; Ludwig Breast Cancer Study Group, 1985; Padmanabhan *et al.*, 1986; Brincker *et al.*, 1987; Tormey *et al.*, 1990). In our study DIA occurred in 75% (166/221) of patients, in 31% of women 40 years or younger and in 94% of those older than 40. Twenty patients (12%) resumed normal menses after temporary DIA. Although temporary DIA could be the expression of a partial suppression of ovarian function, and thus associated with a different prognosis, we did not find a significant difference in DFS among amenorrheic patients with temporary and permanent DIA. However, this result might be affected by the low number of patients who resumed menses. Only the Ludwig Breast Cancer Study Group (1985) dealt with this question, with similar results.

Finally, the time dependent nature of amenorrhea might somehow affect the correlation between DIA and DFS (Anderson *et al.*, 1983). This potential bias, however, is probably small because of the general early occurrence of cessation of menses following initiation of therapy. To overcome this bias, in our study, we employed the Mantel-Byar procedure (Mantel & Byar, 1974) in univariate analysis and the Cox proportional hazard regression model (Cox, 1972) in which amenorrhea is a time varying covariate, as it was done in the ECOG study (Tormey *et al.*, 1990).

The relationship between DIA and length of DFS was analysed in several studies with conflicting results.

In the Milan trial (Bonadonna *et al.*, 1985) only patients younger than 41 were analysed for DFS as related to DIA, since only two patients older than 40 maintained normal menses. Thus, analysis was carried out on a small number of cases, i.e. 19 non amenorrheic and 13 amenorrheic patients, and the conclusion was that DFS was not affected by DIA. The 10 year DFS was 31.6% and 37.2% for the two groups, respectively. In addition, the same authors reported that salvage castration at first relapse induced a higher response rate in patients who had experienced DIA as compared to those who had not (30% *vs* 20% response rate). Based upon these findings they concluded that DIA is not equivalent to complete ovarian failure; this opinion, based on a low number of events, is contrasting with the data of others who assessed ovarian function by measuring levels of circulating sexual hormones during adjuvant chemotherapy (Koyama *et al.*, 1977; Fisher *et al.*, 1979; Rose & Davis, 1980; Ludwig Breast Cancer Study Group, 1985; Padmanabhan *et al.*, 1987), including ourselves (Delrio *et al.*, 1986).

The National Surgical Adjuvant Breast Project group (Fisher *et al.*, 1979) reported a lack of association of DFS with depressed ovarian function induced by melphalan. Since improvement of DFS was slightly better in younger patients, who had lower incidence of DIA, as compared to older women, it was concluded that ovarian suppression could not have played a role. However, no analysis was carried out to evaluate the real relationship between occurrence of DIA and effect of therapy.

By contrast, the Ludwig Group's Trial I (1985) showed that amenorrhea induced by CMF-containing regimens was significantly associated with a longer disease-free survival, in younger patients, in patients who received lower CMF doses and in patients with ER + tumours; it was, thus, suggested that chemotherapy might influence tumour growth by suppression of ovarian endocrine function, besides exerting a direct cytotoxic effect.

Similarly, in the ECOG trial (Tormey *et al.*, 1990) patients developing DIA during adjuvant therapy with CMF, CMFP or CMFPT had a highly significant better survival.

The Danish Breast Cancer Cooperative Group (Brincker *et al.*, 1987) found, in a prospective randomised trial, that cyclophosphamide alone was effective in improving DFS only in patients who experienced DIA, differently from CMF which was active in DIA as well as in NA patients. It was suggested that the effect of chemotherapy could be mediated partly through ovarian suppression and partly through a purely cytotoxic mechanism, the latter being more evident when combination chemotherapy (CMF) was used.

Finally, in 1986, the Manchester group (Padmanabhan *et al.*, 1986) reported, in a randomised trial comparing CMF with observation, that a significant improvement of 3-year DFS and OS was seen only in premenopausal patients who experienced CMF induced permanent amenorrhea: DFS of CMF-treated non amenorrheic patients was similar to that of untreated controls. However, recently the same group reported in abstract form (Richards *et al.*, 1990) that, at the 8-year update of the study, the importance of DIA, in patients younger than 41, has lessened. Since a full-length paper is not available, it is difficult to discuss this result.

Our data show the existence of a significant correlation between DIA and prolongation of DFS. The prognostic relevance of DIA is independent of number of positive nodes, tumour size and number of CMF cycles. These results are consistent with those reported by the majority of studies that could adequately document amenorrhea.

Although the existence of a strong statistical association does not necessarily mean that DIA is causally related to a better prognosis in early breast cancer, and amenorrhea might only be a marker of a greater chemotherapy-induced tumour cell kill (International Breast Cancer Study Group, 1990), our results strongly suggest that the suppression of ovarian function could play a relevant role in the mechanism of action of adjuvant cytotoxic therapy in premenopausal breast cancer patients.

The beneficial effect of oophorectomy in both advanced and early disease, the recent results with LHRH analogues, which produce complete suppression of ovarian function, in patients with advanced disease, the relationship between DIA and improved DFS could suggest that adjuvant hormonotherpay, e.g. LHRH superagonist ± Tamoxifen, might have some effect in premenopausal patients. However, there is no evidence that the effect of endocrine therapies will be as large as chemotherapy. In fact, a recent paper of the International Breast Cancer Study Group (Goldhirsch *et al.*, 1990) shows that chemotherapy provides additional cytotoxic effect over and above those attributable to endocrine mechanisms alone. Whether endocrine therapies can replace chemotherapy for subset of premenopausal women with presumably hormone-responsive primaries is a matter for future clinical trials.

We thank Dr G. Mezzanottea and Dr G. Valsecchi of the Institute of Medical Statistics and Biometry, University of Milan, Italy, for supplying the Mantel-Byar procedure software.

We also thank Dr Richard D. Gelber of the Department of Biostatistics, Harvard School of Public Health, Boston, USA, and Dr Michael K. Palmer, biometrics team leader of the ICI-Pharma for kindly reviewing the manuscript and for their very pertinent comments.

This work was supported by grants (86.00315.44, 87.01188.44, 88.00530.44) from the Italian 'Consiglio Nazionale delle Ricerche', Finalized Project 'Oncologia' and from the 'Associazione Italiana per la Ricerca sul Cancro' (AIRC).

### References

ANDERSON, J.R., CAIN, K.C. & GELBER, R.D. (1983). Analysis of survival by tumor response. *J. Clin. Oncol.*, **1**, 710.

BIANCO, A.R., DE PLACIDO, S., GALLO, C. & 5 others (1988). Adjuvant therapy with tamoxifen in operable breast cancer. *Lancet*, **ii**, 1095.

BMDP STATISTICAL SOFTWARE (1988). Dixon, W.J. (ed.) University of California Press, Los Angeles.

BONADONNA, G., VALAGUSSA, P., ROSSI, A. & 4 others (1985). Ten-year experience with CMF-based adjuvant chemotherapy in resectable breast cancer. *Breast Cancer Res. Treat.*, **5**, 95.

BRINCKER, H., ROSE, C., RANK, F. & 5 others (1987). Evidence of a castration-mediated effect of adjuvant cytotoxic chemotherapy in premenopausal breast cancer. *J. Clin. Oncol.*, **5**, 1771.

BRYANT, A.J.S. & WEIR, J.A. (1981). Prophylactic oophorectomy in operable instances of carcinoma of the breast. *Surg. Gynecol. Obstet.*, **153**, 660.

COX, D.R. (1972). Regression models and life tables. *J. R. Stat. Soc. B*, **34**, 187.

DELRIO, G., DE PLACIDO, S., PAGLIARULO, C. & 10 others (1986). Hypothalamic – pituitary – ovarian axis in women with operable breast cancer treated with adjuvant CMF and tamoxifen. *Tumori*, **72**, 53.

EARLY BREAST CANCER TRIALISTS' COLLABORATIVE GROUP (1988). Effects of adjuvant Tamoxifen and of cytotoxic therapy on mortality in early breast cancer. *N. Engl. J. Med.*, **319**, 1681.

EDITORIAL (1989). Adjuvant systemic treatment for breast cancer meta-analysed. *Lancet*, **i**, 80.

FISHER, B., SHERMAN, B., ROCKETTE, H., REDMOND, C., MARGOLESE, R. & FISHER, E.R. (1979). l-Phenylalanine mustard (L-PAM) in the management of premenopausal patients with primary breast cancer. Lack of association of disease-free survival with depression of ovarian function. *Cancer*, **44**, 847.

GOLDHIRSCH, A., GELBER, R.D & CASTIGLIONE, M. (1990). The magnitude of endocrine effects of adjuvant chemotherapy for premenopausal breast cancer patients. *Ann. Oncol.*, **1**, 183.

INTERNATIONAL BREAST CANCER STUDY GROUP (1990). Late effects of adjuvant oophorectomy and chemotherapy upon premenopausal breast cancer patients. *Ann. Oncol.*, **1**, 30.

KAPLAN, E.L. & MEIER, P. (1958). Nonparametric estimation from incomplete observation. *J. Am. Stat. Assoc.*, **53**, 457.

KOYAMA, H., WADA, T., NISHIZAWA, Y. & 6 others (1977). Cyclophosphamide-induced ovarian failure and its therapeutic significance in patients with breast cancer. *Cancer*, **39**, 1403.

LUDWIG BREAST CANCER STUDY GROUP (1985). A randomized trial of adjuvant combination chemotherapy with or without prednisone in premenopausal breast cancer patients with metastases in one to three axillary lymph nodes. *Cancer Res.*, **45**, 4454.

MANTEL, N. (1966). Evaluation of survival data and two new rank order statistics arising in its consideration. *Cancer Chem. Rep.*, **50**, 163.

MANTEL, N. & BYAR, D.P. (1974). Evaluation of response-time data involving transient states: an illustration using heart transplant data. *J. Am. Stat. Assoc.*, **69**, 81.

MEAKIN, J.W., ALLT, W.E.C., BEALE, F.A. & 10 others (1983). Ovarian irradiation and prednisone following surgery and radiotherapy for carcinoma of the breast. *Breast Cancer Res. Treat.*, **3** (Suppl. 1), 45.

NISSEN-MEYER, R. (1967). The role of prophylactic castration in the therapy of human mammary cancer. *Eur. J. Cancer*, **3**, 395.

PADMANABHAN, N., HOWELL, A. & RUBENS, R.D. (1986). Mechanism of action of adjuvant chemotherapy in early breast cancer. *Lancet*, **ii**, 411.

PADMANABHAN, N., WANG, D.Y., MOORE, J.W. & RUBENS, R.D. (1987). Ovarian function and adjuvant chemotherapy for early breast cancer. *Eur. J. Cancer Clin. Oncol.*, **23**, 745.

RICHARDS, M.A. O'REILLY, S.M., HOWELL, A. & RUBENS, R.D. (1990). Adjuvant CMF in node positive breast cancer: Guy's/Manchester trial at 8 years. *Proc. Am. Soc. Clin. Oncol.*, **9**, 18.

ROSE, D.P. & DAVIS, T.E. (1980). Effects of adjuvant chemohormonal therapy on the ovarian and adrenal function of breast cancer patients. *Cancer Res.*, **40**, 4043.

SIMON, R. & MAKUCH, R.W. (1984). A non-parametric graphical representation of the relationship between survival and the occurrence of an event: application to responder versus non-responder bias. *Stat. Med.*, **3**, 35.

TORMEY, D.C., GRAY, R., GILCHRIST, K. & 5 others (1990). Adjuvant chemohormonal therapy with cyclophosphamide, methotrexate, 5-fluorouracil, and prednisone (CMFP) or CMFP plus tamoxifen compared with CMF for premenopausal breast cancer patients. An Eastern Cooperative Oncology Group Trial. *Cancer*, **65**, 200.